# Superior Mesenteric Artery Thrombosis Following a Snakebite: A Rare Case

**DOI:** 10.7759/cureus.111082

**Published:** 2026-06-18

**Authors:** Ravi S, Spoorthy Srinivas, Manjunath B D

**Affiliations:** 1 Department of General Surgery, Bangalore Medical College and Research Institute, Bengaluru, IND

**Keywords:** mesenteric ischemia, sma thrombosis, snakebite, systemic manifestations of viper bite, viper bite

## Abstract

Snakebites are common emergencies in tropical countries, usually presenting with hematotoxic, neurotoxic, or myotoxic effects. Vascular thrombosis as a systemic complication is extremely rare. We report a case of superior mesenteric artery (SMA) thrombosis following a viper bite, leading to acute mesenteric ischemia. A 39-year-old male patient presented with abdominal pain, vomiting, loose stools, and reduced urine output following a snakebite with *Daboia russelii *to the right index finger. He had received 20 vials of anti-snake venom at a peripheral hospital. On examination, he was tachycardic with abdominal distension, diffuse tenderness, guarding, and rigidity. Blood investigations suggested leukocytosis, thrombocytopenia, and elevated creatinine levels. Imaging revealed dilated small bowel loops and near-complete thrombosis of the jejunal and ileal branches of the SMA. The patient underwent exploratory laparotomy, with resection of gangrenous small bowel, with double-barrel stoma. Despite emergent surgical intervention and intensive supportive care, the patient’s condition deteriorated rapidly. The patient succumbed to multi-organ failure and died on postoperative day two. This case highlights the rare but lethal complication of acute mesenteric ischemia following viper envenomation. Early recognition and timely surgical intervention are critical for favorable outcomes.

## Introduction

Snakebite envenomation is a critical yet frequently neglected tropical disease resulting from accidental exposure to venomous snakes. It remains a significant global public health challenge, particularly across tropical and subtropical regions. According to World Health Organization (WHO) data, there are approximately 5.4 million snakebites annually, with 1.8 to 2.7 million cases resulting in clinical envenomation [1]. These incidents lead to an estimated 81,000 to 138,000 deaths. India bears a disproportionate burden, accounting for approximately 1.2 million snakebite deaths over a 20-year period, averaging 58,000 fatalities annually. However, the true scale of this crisis is likely underreported, as only a small fraction of victims reach tertiary care, and only 23% of snakebite-related deaths in India occur within a hospital setting [2].

The Russell’s viper (*Daboia russelii*) is one of the “Big Four” medically significant snake species in India. Clinical envenomation typically begins with intense local pain and rapidly progressing edema of the affected extremity. Systemic manifestations often involve coagulopathy, characterized by gingival bleeding, hematuria, and hemoptysis. While local tissue necrosis is generally superficial, severe cases can progress to deep muscle involvement and extensive blistering. Acute kidney injury (AKI), severe disseminated intravascular coagulation (DIC), cellulitis, gangrene, and hypotension are the dreadful complications of viper envenomation [3].

While the localized effects and nephrotoxicity of Russell’s viper venom are well-established, mesenteric ischemia remains a poorly documented complication in the existing literature. The venom contains a complex mixture of procoagulant enzymes, phospholipases, and metalloproteinases capable of inducing endothelial injury, systemic DIC, and vasospasm. Although hemorrhagic manifestations are the hallmark of viperine bites, thrombotic events, specifically superior mesenteric artery (SMA) thrombosis, are exceedingly rare. The hypothesized mechanisms for such events include venom-induced direct endothelial damage, potent prothrombotic activity, and profound vasospasm [4].

SMA thrombosis is an atypical, life-threatening complication that often manifests as acute mesenteric ischemia. Due to its non-specific clinical presentation and frequent delays in seeking medical attention, it poses a formidable diagnostic challenge for the medical community. We present a case of mesenteric ischemia following a Russell’s viper bite (*Daboia russelii*) in a 39-year-old male from rural Kolar, Karnataka. To our knowledge, this is a unique and inaugural case report of its kind from the Indian subcontinent.

## Case presentation

A 39-year-old male with no comorbidities and no adverse habits presented to our tertiary care center in Bengaluru, Karnataka, eight days after sustaining a snakebite from* Daboia russelii *to his right index finger while working on his farm in Kolar, Karnataka. Immediately following the injury, the patient received primary care at a local facility, which included 20 vials of polyvalent anti-snake venom, intravenous metronidazole, and analgesics. Due to localized swelling and signs of compartment syndrome, a fasciotomy of the right index finger was performed on the day of the bite. He was subsequently referred to our center following the development of multi-organ dysfunction syndrome.

Three days after the snakebite, the patient developed progressive gastrointestinal symptoms, including continuous colicky abdominal pain involving all quadrants; four to six episodes of non-projectile, non-bilious vomiting; and four to five episodes of loose stools per day. These symptoms were accompanied by significant fatigue and a noticeable reduction in urine output.

​Upon physical examination, the patient was tachycardic (heart rate: 114 beats/minute) and tachypneic (respiratory rate: 20 cycles/minute). Abdominal examination revealed distension and diffuse tenderness, with associated guarding and rigidity. The fasciotomy wound on the right index finger appeared healthy with no signs of secondary infection. Laboratory investigations confirmed systemic envenomation, notable for leukocytosis, thrombocytopenia, and AKI, as shown in Table 1. All investigations were traced from the day of snakebite envenomation.

**Table 1:** Blood investigation parameters.

Parameters	Normal range	Day 1	Day 3	Day 5	Day 7	Day 8
Hemoglobin (g/dL)	13–15	14.1	13.9	10.6	10.1	10.2
Red blood cell count (million/mm^3^)	4.5–5.5	5.14	5.08	3.89	3.56	3.53
Packed cell volume (%)	40–50	41.3	41.5	31.5	30.8	31
White blood cell count (cells/mm^3^)	4,000–12,000	18,390	16,310	19,037	24,568	25,897
Platelets (/L)	150–450 × 10^9^	48 × 10^9^	75 × 10^9^	75 × 10^9^	50 × 10^9^	52 × 10^9^
Sodium (mEq/L)	135–145	139	138	135	140	136
Potassium (mEq/L)	3.5–5	4.5	4.6	4.5	4.7	4.8
Urea (mg/dL)	16.6–48.5	61	94	107	110	108
Creatinine (mg/dL)	0.7–1.3	2.2	1.8	1.5	2.2	2.3
Total bilirubin (mg/dL)	<1.2	1.5	1.1	1.3	1.2	1.3
Serum albumin (mg/dL)	3.2–4.8	3.7	3.4	2.8	2.5	2.5
Prothrombin time (seconds)	9–14	20.2	-	21	-	20
International normalized ratio	0.9–1.1	1.6	-	1.5	-	1.5
Creatine phosphokinase (U/L)	30–200	-	399	-	279	-
Urine albumin	-	-	-	-	Present	-

​Appropriate resuscitative measures were undertaken to stabilize the patient. An erect abdominal X-ray revealed multiple air-fluid levels (Figure 1) within the small bowel, and ultrasonography confirmed mild ascites with dilated, hypoperistaltic prominent bowel loops. Contrast-enhanced computed tomography (CECT) of the abdomen demonstrated near-complete thrombosis of the jejunal and ileal branches of the SMA, hypoenhancement of the proximal and mid-ileal loops, and mild free intraperitoneal fluid (Figure 2).

**Figure 1:**
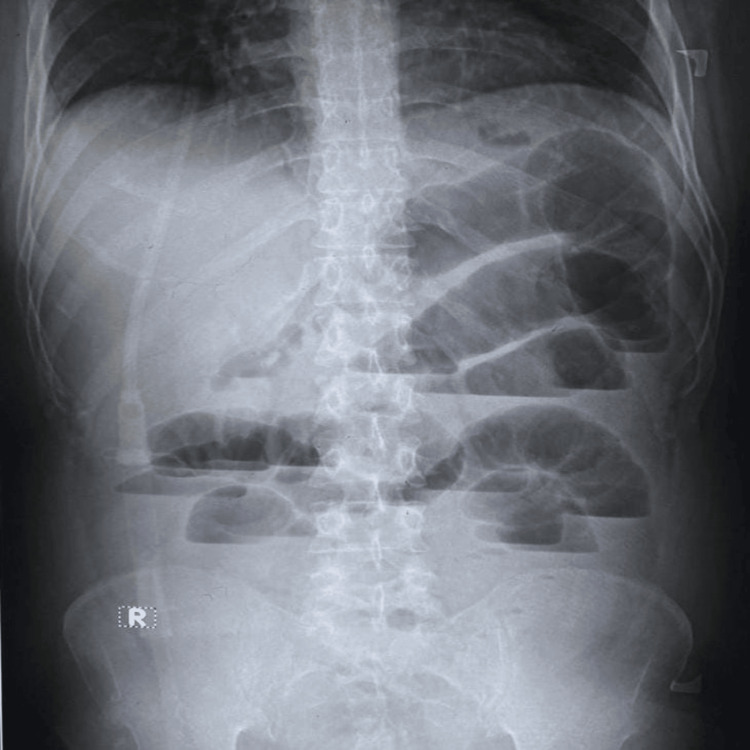
Erect X-ray of the abdomen depicting multiple air-fluid levels with dilated bowel loops.

**Figure 2:**
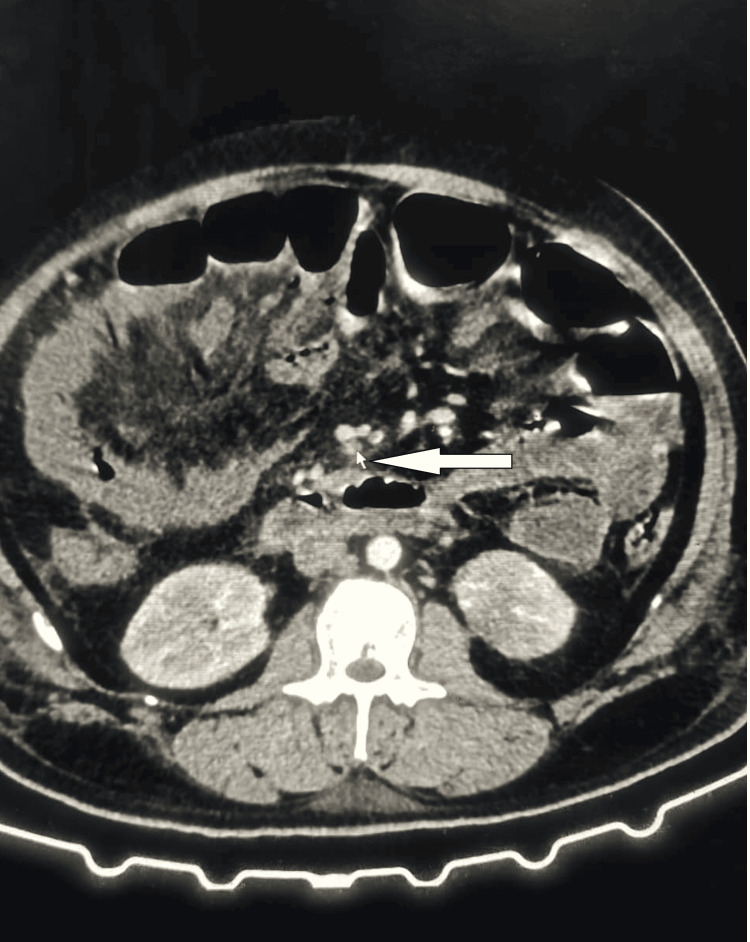
Axial constrast-enhanced computed tomography image showing near-complete thrombosis of the jejunal/ileal superior mesenteric artery branches with bowel hypoenhancement. The white arrow depicts no contrast opacification in the jejunal and ileal branches of the superior mesenteric artery.

​Following a vascular surgery consultation, the patient underwent an emergency exploratory laparotomy under general anesthesia. Intraoperative findings included 1,000 mL of feculent fluid and extensive gangrenous bowel extending from 100 cm distal to the duodenojejunal (DJ) junction to 50 cm proximal to the ileocecal junction. A jejunal perforation was identified 80 cm from the DJ junction, associated with clumped and indurated omentum (Figure 3). Figure 4 depicts gangrenous bowel loops. The gangrenous segment was resected, and a double-barrel stoma was fashioned with the placement of bilateral abdominal drains. The patient underwent an exploratory laparotomy with resection of gangrenous small bowel with peritoneal lavage and double-barrel stoma. The patient was shifted to the intensive care unit in view of persistent acidosis and hypotensive shock.

**Figure 3:**
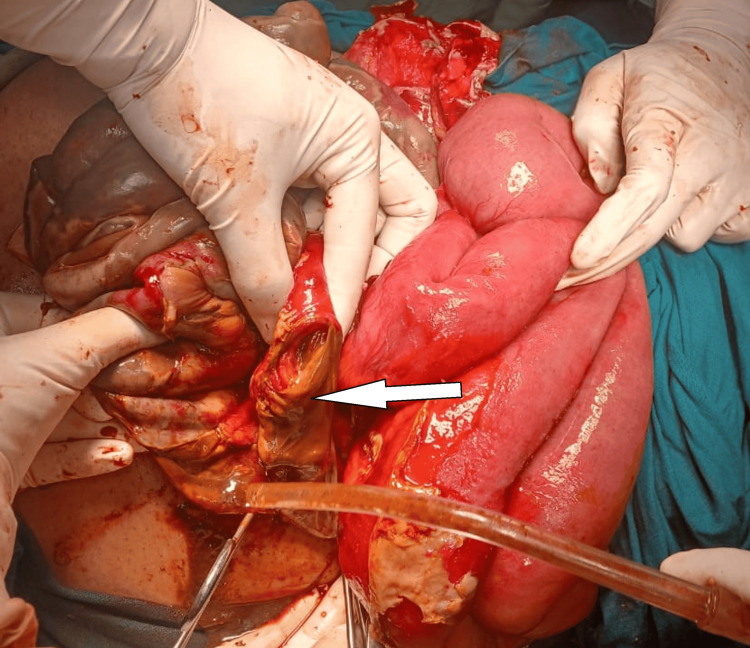
Jejunal perforation noted 80 cm from the duodenojejunal junction. The white arrow depicts the distal side of jejunal perforation 80 cm from the duodenojejunal junction.

**Figure 4:**
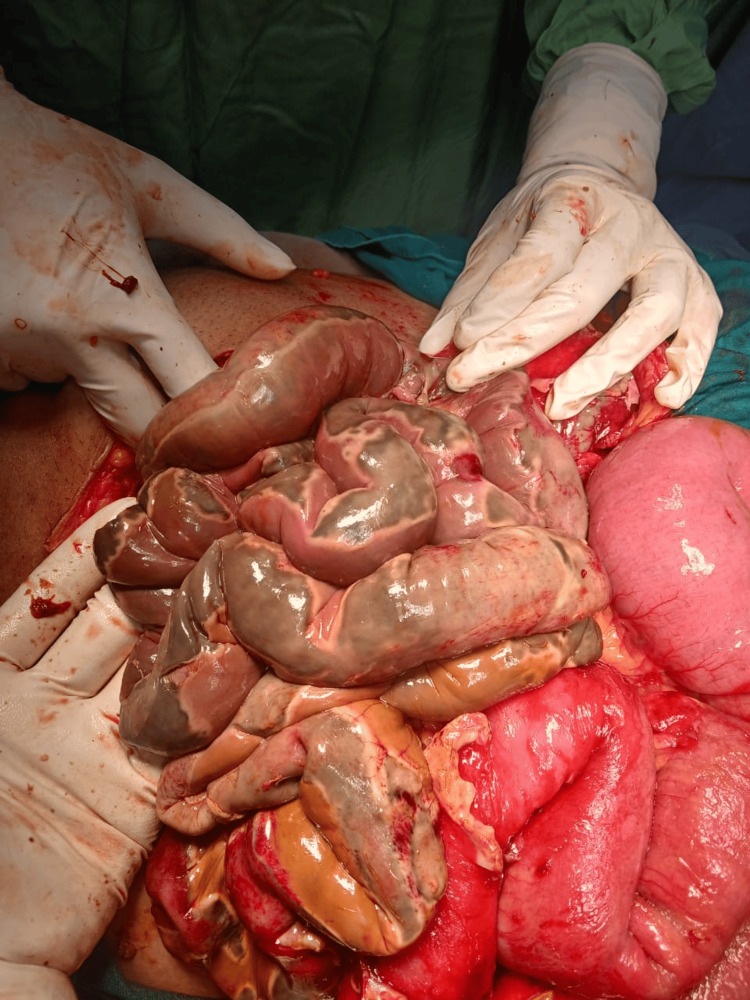
Gangrenous small bowel 100 cm of the duodenojejunal junction to 50 cm from the ileocecal junction.

Despite aggressive surgical intervention for the identified mesenteric ischemia, the patient succumbed to refractory septic shock and multi-organ failure on postoperative day two. Histopathological examination of the resected specimen confirmed transmural necrosis consistent with peritonitis. Figure 5 depicts a microscopic image of the resected bowel loops with dense mixed inflammatory infiltrates with edema, congestion, and necrosis, suggestive of gangrenous bowel. Postmortem examination confirmed the primary insult, revealing two distinct puncture wounds on the right index finger. Internal findings revealed that the peritoneal cavity and serosal surfaces of the intestines were extensively smeared with pus, indicating generalized suppurative peritonitis secondary to the ischemic bowel perforation. This systemic inflammatory state was further corroborated by the presence of bilateral straw-colored pleural effusions. Notably, the discovery of purulent material within the brain suggested a terminal hematogenous seeding of the infection.

**Figure 5:**
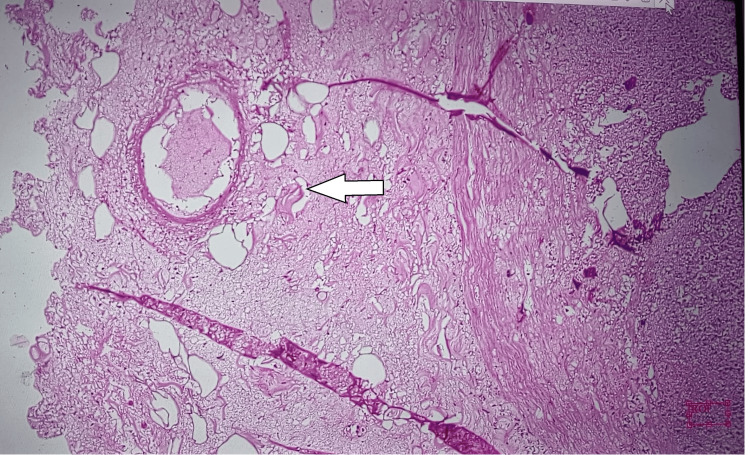
Microscopic image of the resected bowel loops with dense mixed inflammatory infiltrates with edema, congestion, and necrosis, as depicted by the white arrow, suggestive of gangrenous bowel.

## Discussion

​The pathophysiology of Russell’s viper envenomation is traditionally characterized by a “hemorrhagic syndrome” driven by metalloproteinases and phospholipases that disrupt the vascular endothelium and consume clotting factors [4]. However, the venom also contains potent procoagulant enzymes, such as Factor X and prothrombin activators, which can paradoxically trigger systemic microvascular thrombosis and large-vessel occlusion. While localized tissue necrosis and AKI are common, the occurrence of mesenteric ischemia secondary to SMA thrombosis remains an exceedingly rare clinical phenomenon. Such events are often under-recognized due to the overshadowing nature of systemic bleeding and renal failure. A review of the global literature revealed only a handful of documented cases involving visceral infarction following viperine or crotaline bites.

Rosenthal et al. [5] were among the first to report a case of intestinal ischemia following a snakebite, specifically involving* Lachesis muta* in Costa Rica. In their report, a 64-year-old patient was initially discharged after three days but returned shortly thereafter with severe abdominal pain. Investigations revealed an obstruction of the SMA, which had led to extensive necrosis of the ileum and cecum, necessitating urgent surgical intervention.

Similarly, Galan et al. presented a case of acute mesenteric ischemia following a bite from a lancehead (*Bothrops*) snake in the Northern Brazilian Amazon [6]. Although the patient initially exhibited typical features of envenomation, including local edema and coagulopathy, the sudden onset of severe abdominal pain shifted the clinical focus. Imaging confirmed reduced intestinal perfusion due to venom-induced thrombosis. This case underscored the life-threatening nature of thrombotic complications in *Bothrops* envenomation and emphasized the necessity of early recognition and aggressive antivenom therapy.

In North Africa, Mourouth et al. described a case of a 66-year-old patient who suffered a rare combination of lower limb ischemia and mesenteric infarction following a horned viper bite [7]. The authors highlighted that while viper venom is primarily hemotoxic, its procoagulant proteins can trigger catastrophic arterial thromboses. Despite the administration of FAV-Afrique antivenom and emergency intestinal resection for extensive small bowel obstruction, the patient’s clinical course was complicated by DIC, rhabdomyolysis, and acute renal failure, ultimately leading to death from refractory septic shock.

Furthermore, Beer et al. documented a case of* Vipera aspis* envenomation characterized by a complex presentation of shock, neurotoxicity, and myoglobinuria [8]. In this instance, the patient developed iliac vessel thrombosis and subsequent intestinal infarction, requiring a right hemicolectomy. These global cases collectively illustrate that while the species may vary, the underlying mechanism of venom-induced consumption coagulopathy can manifest as focal arterial thrombosis in diverse geographical contexts.

Acute mesenteric ischemia remains a diagnostic challenge because it frequently presents with non-specific symptoms that may be mistaken for the general systemic distress of envenomation. CECT remains the imaging modality of choice for early detection. Timely surgical intervention is the only definitive measure to prevent high mortality rates. This case serves as a critical reminder for clinicians to remain vigilant when a snakebite victim reports persistent abdominal pain, even if traditional markers of coagulopathy appear to be stabilizing.

Ultimately, this case demonstrates that snakebite management must evolve to include a high index of suspicion for atypical thrombotic events. In our patient, the confluence of delayed presentation and non-specific gastrointestinal symptoms disguised a catastrophic SMA thrombosis until multi-organ dysfunction had already stabilized, illustrating the narrow window for survival. The transition from a treatable envenomation to an irreversible ischemic event can be swift and silent; therefore, recognizing abdominal pain as a surgical “red flag” in viperine bites is not merely a diagnostic nuance, but a life-saving imperative.

## Conclusions

The clinical management of snakebite envenomation is a complex undertaking that extends far beyond the standardized administration of antivenom. As illustrated by this case, systemic outcomes are highly heterogeneous and can manifest in catastrophic vascular events, including mesenteric ischemia. Clinicians must maintain a high index of clinical suspicion when a victim presents with abdominal pain, as this may be the primary indicator of life-threatening visceral infarction rather than benign local effects. Rapid diagnostic imaging and timely surgical intervention are critical components of the care pathway to mitigate morbidity and ensure patient survival.
